# The role of Lactobacillus species in the control of Candida via biotrophic interactions

**DOI:** 10.15698/mic2020.01.702

**Published:** 2019-11-25

**Authors:** Isabella Zangl, Ildiko-Julia Pap, Christoph Aspöck, Christoph Schüller

**Affiliations:** 1 University of Natural Resources and Life Sciences Vienna (BOKU), Department of Applied Genetics and Cell Biology (DAGZ), Tulln, Austria.; 2 University Hospital of St. Pölten, Institute for Hygiene and Microbiology, St Pölten, Austria.; 3 Bioactive Microbial Metabolites (BiMM), BOKU, Tulln, Austria.

**Keywords:** Lactobacillus, Candida, interaction, vaginal microbiome, probiotics

## Abstract

Microbial communities have an important role in health and disease. *Candida* spp. are ubiquitous commensals and sometimes opportunistic fungal pathogens of humans, colonizing mucosal surfaces of the genital, urinary, respiratory and gastrointestinal tracts and the oral cavity. They mainly cause local mucosal infections in immune competent individuals. However, in the case of an ineffective immune defense, *Candida* infections may become a serious threat. *Lactobacillus* spp. are part of the human microbiome and are natural competitors of *Candida* in the vaginal environment. Lactic acid, low pH and other secreted metabolites are environmental signals sensed by fungal species present in the microbiome. This review briefly discusses the ternary interaction between host, *Lactobacillus* species and *Candida* with regard to fungal infections and the potential antifungal and fungistatic effect of *Lactobacillus* species. Our understanding of these interactions is incomplete due to the variability of the involved species and isolates and the complexity of the human host.

## INTRODUCTION

The composition of the complex microbial communities hosted by the human body is highly dependent on the environmental conditions and host factors. Thus, microbiome characteristics vary from site to site and also between individuals. Microbiomes play an important role in pathogen resistance, strengthening the immune system and nutrition uptake [1]. The mycobiome is a subset of the microbiome and reflects the load and composition of fungal cells in the human body [2]. The human vaginal microbiome is associated with prevention of various urogenital diseases such as, bacterial vaginosis, yeast and viral infections, sexually transmitted infections or urinary tract infections [3]. *Lactobacillus* spp. and *Candida* spp. are commensals of the human microbiome [4]. *Candida* is also an opportunistic pathogen and can cause infections like vulvovaginal candidiasis (VVC), as well as more severe systemic infections. About 75% of women suffer from VVC at least once during their lifetime [5]. Systemic *Candida* infections occur in immunocompromised patients [6], caused by *Candida albicans* (about 50%) and *Candida glabrata* (15%-25%) [7–9]. *Lactobacillus* spp. are part of the healthy vaginal microbiome and are seen as promising probiotics to treat or prevent mucosal *Candida* infections or to support traditional treatment options [10]. Probiotics are defined by the WHO as live microorganisms that, when administered or consumed in adequate quantities, confer health benefits [4]. Applying *Lactobacillus* to treat fungal infections is based on the premise that certain *Lactobacillus* species exert a protective effect *in vivo* by reducing the adhesion of the fungus to the vaginal mucosa [11, 12], production of organic acids [11, 13, 14] and favorable metabolites [15–18] as well as enhancing vaginal epithelial cell immune defense mechanisms [19, 20].

Several articles discuss the composition of the human microbiome and mycobiome in detail [21–23] and its importance with regard to vaginal health [24, 25]. The role of *Lactobacillus* species as general probiotics has been assessed previously [4, 26–28]. Here we focus on the molecules and mechanisms behind the putative interactions between *Lactobacillus* and *Candida* spp.

## HUMAN ASSOCIATED *LACTOBACILLI*

Most vaginal microbiota contain *Lactobacillus* species [29–31], with quantity and proportion of specific species and strains varying between women of specific ethnic origins [24]. Alterations in this microbiome often lead to symptomatic conditions, for example bacterial vaginosis or other mucosal vaginal infections [25]. Changes in the quantity of vaginal microorganisms also play a role in septic postpartum, neonatal infections, pelvic inflammatory disease, miscarriage, pre-term birth and increased HIV acquisition and onward transmission [29]. *Lactobacillus* spp. are facultative anaerobe, gram-positive, catalase-negative, non-spore-forming rods. They can produce lactic acid as end product of homolactic fermentation [32]. About 200 species are associated with the Lactobacillus genus complex [33, 34]. Various *Lactobacillus* spp. are part of the normal human gastrointestinal and vaginal flora. However, the species involved differ between gastrointestinal (GI) and vaginal tract. In fact, *Lactobacillus* spp. are the predominant members of the vaginal microbiome in healthy women [26] and are thought to help preventing vaginal dysbiosis [35]. A healthy vaginal environment is often associated with a *L. crispatus*, *L. gasseri* and/or a *L. jensenii* dominated microbiome [36, 37]. Besides, there are also women with a microbiome consisting of higher proportions of facultative and anaerobe bacteria [3], including *Prevotella* or *Gardnerella* [23, 24]. These non-*Lactobacillus* dominated microbiomes are also considered as a healthy, normal vaginal flora in asymptomatic women and are not necessarily a sign for disease.

One of the most frequently isolated organisms in the vaginal tract is *L. iners*. It is found in about 50% of both, healthy and diseased women, which is probably due to its high degree of adaption to the sometimes changing vaginal environment. *L. iners* has a small genome which indicates a parasitic or symbiotic lifestyle. It is associated with increased risk of vaginal dysbiosis [37]. A *L. crispatus* dominated microbiome is the second most common environment. Compared to *L. iners* and mixed vaginal microbiomes, dominance of *L. crispatus* is associated with a more stable microbiome and reduced probability to shift towards bacterial vaginosis [24]. Analysis at the species-level showed a shift from healthy vaginal microbiome dominated by *L. crispatus* to *L. iners* in genital infections such as VVC, *Chlamydia trachomatis* and bacterial vaginosis [38]. The composition of the vaginal microbiome not only differs between individual women, but also by ethnicity [23, 24, 37, 39]. With changing dominating bacteria, also the pH of the vaginal environment changes slightly. The lowest median pH was reached by a *L. crispatus* dominating microbiome (pH 4.0 ± 0.3) and the highest pH was detected in women with a non-*Lactobacillus* dominated one (pH 5.3 ± 0.6) [3]. The vaginal environment also fluctuates throughout the menstrual cycle. During menstruation a slight decrease in *Lactobacillus* spp. and a relative increase in other bacteria occur [24]. This may be explained by the fluctuation in estrogen levels, as high levels of estrogen may favor a *Lactobacillus* dominated environment [40]. Estrogen levels are low at the beginning of the menses, which could have a negative effect on *Lactobacillus* spp. numbers [24]. Preterm delivery is correlated with dysbiosis, lower vaginal levels of *L. crispatus* and higher levels of other taxa [41, 42]. Vitamin D level correlates with the vaginal *L. crispatus* abundance and could thus prevent pregnancy complications [43]. Following the conclusions of Hickey and colleagues it is surprising that since Döderlein's initial discovery and antibiotic activity of human associated lactic acid bacteria about 150 years ago, the microbial ecosystem of the human vagina is still not fully understood [23].

## HUMAN ASSOCIATED FUNGI

Fungi contribute marginally to the human microbiome but nobody is fungus-free. In the gut, about 0.1% of the species are of fungal origin according to metagenomics studies [44, 45]. Nevertheless, fungi produce unique metabolites and enzymes and thus the fungal constituents may help maintaining microbial community structure, metabolic function and immune-priming frontiers [46]. A small number of fungal species are asymptomatic colonizers like *Candida spp.*, *Malassezia spp.*, *Cryptococcus neoformans* or *Pneumocystis jirovecii.* They have the potential to become pathogenic for example when the host is immunocompromised or the host environment is disturbed by antibiotic treatment [47]. Despite antifungal therapy they survive as persisters [48] or acquire transient antifungal resistance (heteroresistance) [49].

*Candida* spp. are the fourth most common cause of nosocomial systemic infections in the United States. *C. albicans* has the highest prevalence in humans [50]. It is part of the oral, gut and vaginal mucous microbiota and is associated with causing VVC [10, 47]. Other relevant human associated species are *C. glabrata*, *C. tropicalis*, *C. krusei*, *C. parapsilosis, C. dubliniensis* and *C. lusitaniae* [51–53].

### Candida albicans

*C. albicans* can be isolated in up to 80% of healthy individuals and has its natural habitat on skin and mucous membranes like oral or vaginal epithelium and urogenital tract [50]. *C. albicans* can colonize without symptoms host niches which differ in nutrient availability, pH or CO_2_ levels. Its ability to thrive in these conditions is an indication for its commensalism in humans and an important feature for its pathogenicity as well. *C. albicans* is pleomorphic and able to grow as yeast, as pseudohyphae cells or as true hyphae [21, 50, 54]. Pathogenicity is associated with invasive hyphal growth [55], whereas commensalism of *C. albicans* happens mostly in the adherent yeast cell form (also called blastospores), since epithelial cells fail to efficiently recognize them [54, 56]. *C. albicans* blastospores are associated with vaginal transmission [5]. *C. albicans* belongs to the CTG clade of Ascomycota and Saccharomycotina [57, 58] with many associated asexual species [59]. CTG addresses the reassignment of the conventional Leu CUG codon to serine [60, 61]. The selective advantage of such as reassignment is speculative but might cause a higher variability of surface exposed proteins to adapt to environmental challenges such as recognition by the immune system [62].

*C. albicans* is the cause of most oral and vaginal Candidiasis [10]. Important key virulence factors of *C. albicans* include biofilm formation, countering host innate immunity, evasion from host immune system, adherence to host surface, yeast to hyphae transition and production of candidalysin [50, 54, 63]. Candidalysin is a cytolytic peptide toxin mainly expressed by hyphal cells that directly damages epithelial membranes leading to activation of a danger response signaling pathway and thus epithelial immunity [64]. Another important virulence factor is the ability to escape from phagocytosis by neutrophils (the major fungal killing effector immune cells) and macrophages. C. *albicans* escapes by inducing non-lytic expulsion, increasing the alkalinity, hyphae formation, generating protective antioxidants or induction of pyroptosis to lyse the phagocyte [50].

Furthermore, biofilm formation is important for pathogenicity and treatment, because biofilms, among other traits, exhibit higher antifungal resistance compared to planktonic cells. Only two classes of agents, amphotericin B and echinocandins were found to have an *in vitro* efficacy against fungal biofilms [65]. Additionally, mechanisms of the immune system against infections, such as macrophage migration towards *C. albicans* are reduced when cells are in a biofilm structure [66]. Biofilm formation in *C. albicans* develops in several stages. After initial adhesion and biofilm arrangement, the biofilm disperses [67, 68]. These dispersed biofilm cells were shown to build more robust biofilms and exhibit a higher virulence [68]. Mixed species biofilms are the basis for intimate contacts and cross kingdom interactions between bacteria and fungi.

### Non-albicans *Candida* species

Recent studies show that isolation of non-*albicans Candida* (NAC) species became more frequently isolated in the last two decades [69–72]. This is perhaps due to the better treatment and thus lower incidence of *C. albicans*. The most important strains associated with diseases are *C. glabrata*, *C. tropicalis*, *C. krusei*, *C. dubliniensis* and *C. parapsilosis* [73]. Susceptibility to antifungal drugs differs between the species. *C. glabrata* and *C. krusei* are intrinsically resistant to azoles, *C. parapsilosis* to echinocandins [74], and *C. auris* an emerging species is notably resistant to several drugs [75, 76].

*C. glabrata,* the most frequent isolated NAC species in Europe and North America, is isolated in around 10% of candidiasis patients [70, 73]. *C. glabrata* is related to the bakers' yeast *Saccharomyces cerevisiae* [8], and belongs to the Nakaseomyces clade [77]. Similar to *S. cerevisiae, C. glabrata* grows only in yeast form. Detailed genomic analyses show the divergence of the *C. glabrata* isolates into several distinguishable clades and document remnants of occasional mating events [78–80]. Other human pathogenic *Candida* species like *C. tropicalis* or *C. dubliniensis* are closer related to *C. albicans* [81]. The phylogenetic distance of pathogenic *Candida* spp. suggests pathogenicity has evolved independently [9]. Human virulence of *Candida* species has developed in several independent ways and entails different mechanisms regarding adhesion, persistence, immune system evasion, stress resistance, and nutrient requirements [82–84]. *C. glabrata* has highly efficient adhesion to various surfaces due to a range of adhesins [85], high stress resistance and in addition has the shortest replication time of all *Candida* spp tested so far (our unpublished results) [86].

*C. glabrata* strains have an intrinsic resistance to azole antifungal drugs [87, 88]. *C. glabrata* does not cause epithelial damage and does not provoke a strong immune response. Furthermore, it can reside in macrophages without immediately harming them [9]. *C. glabrata* does not form a biofilm on vaginal mucosa in a mouse model. However, it is able to form biofilms on abiotic surfaces such as medical devices such as vascular and urinary catheters [89, 90]. Biofilms on abiotic surfaces consist of yeast cells in multilayer structures [91]. Interestingly, a positive interaction between *C. albicans* and *C. glabrata* for host infection has been suggested. Mixed biofilms consisting of *C. glabrata* and *C. albicans* lead to more robust and complex structures and improve antifungal resistance [92]. We observed relatively frequent co-isolation of both fungi. Furthermore, *C. albicans* and *C. glabrata* co-infection seems also to be important for both initial colonization and establishment of oropharyngeal candidiasis infection by *C. glabrata* [93].

Other NCAs are such as the *C. parapsilosis* complex, *C. tropicalis*, are, with regional differences, of more or less of similar prevalence as *C. glabrata*, while *C. dubliniensis*, *C. krusei* and *C*. *lusitaniae* are less frequently isolated [88, 94]. In general, commensal and pathogenic *Candida* species are confronted with and are part of the microbiome. Thus, multiple interactions, either synergistic or antagonistic, with various bacterial species are common [95].

## VULVOVAGINALE CANDIDIASIS

The most common classic mucosal vaginal infections include bacterial vaginosis (BV), Trichomoniasis and VVC [96]. VVC is an acute inflammatory disease and one distinguishes between the uncomplicated and complicated form [40]. Symptoms for both are acute pruritus, erythematous vulva, dyspareunia and white vaginal discharge, which makes both variants clinically indistinguishable [5]. Complicated VVC is defined as a recurrent infection or infections in pregnant, immunocompromised and debilitated persons, as well as infections caused by *Candida* species other than *C. albicans* [40]. Uncomplicated VVC comprises infections with *C. albicans*, non- recurrent infections and/or infections in an immunocompetent host [40]. Around 11-30% of VVC are induced by NAC species [6]. A recurrent form of VVC develops in about 5-8% of the cases [5, 97]. Around 15% of recurrent VVC infections are induced by *C. glabrata* [40]. Diagnosis of vaginitis is normally conducted by microscopy, wet mount, culture or PCR [96, 98].

Treatment options for VVC comprise a variety of antifungal agents, e.g. fluconazole (oral), miconazole (topical) or clotrimazole (topical) [40]. Both, oral and topical antifungal agents are prescribed for 1 to 7 days, depending on dosage and drug [5]. Fluconazole is the preferred choice against fungal infections as it can be taken orally in single dose [5]. Complicated VVC often needs a more rigid regime in order to keep the vaginal fungal load at reduced levels [10]. In addition, some NAC species are intrinsically resistant or less susceptible [10, 99]. In that case a broader spectrum agent like amphotericin B deoxycholate, voriconazole, or echinocandins, such as caspofungin and anidulafungin, can be used to treat the infection [7, 10]. Infections caused by *C. glabrata* can also be treated alternatively with boric acid or flucytosine [100]. Acquired antimycotic resistance emerges mainly during treatment due to selection in patients and is usually confined and rarely transferred between patients [74]. Still, therapy improvements for species with antimycotic resistance are needed.

## METABOLIC BYPRODUCTS OF *LACTOBACILLUS*

**Figure 1:**
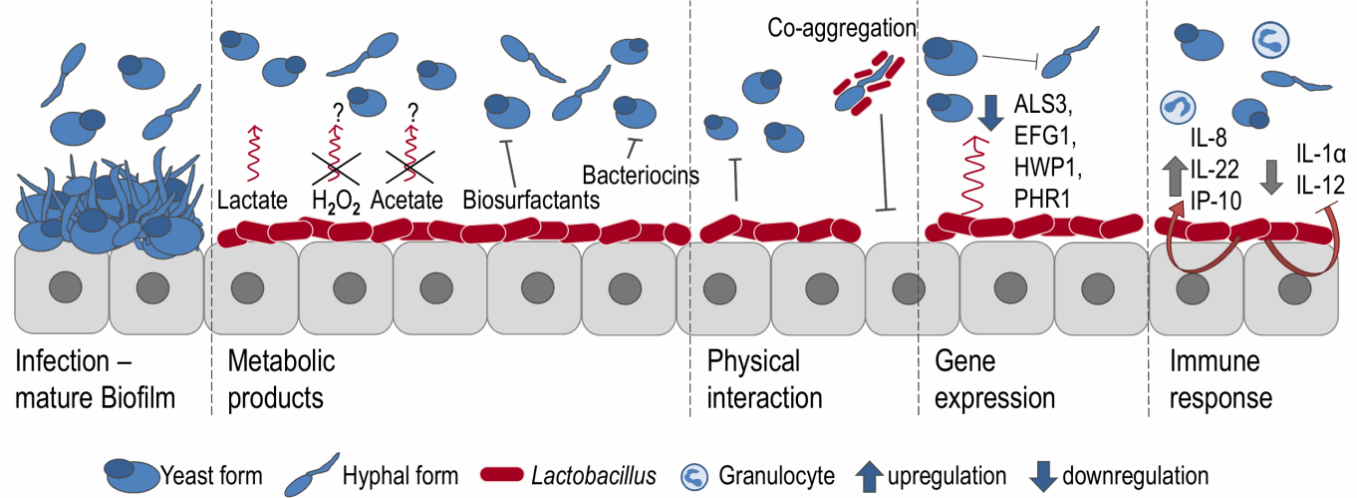
FIGURE 1: Interactions between *C. albicans* and *Lactobacillus* spp. Production of metabolic products prevent colonization through *C. albicans* either due to prevention of adhesion to the epithelial cell wall, or due to high concentrations of organic acids, exerting a fungistatic effect. H_2_O_2_ and acetate probably don't play a role in the vaginal tract. Saturation of adhesion sites and co-aggregation of *Lactobacillus* spp. prevent adherence of *Candida* spp. Gene expression in *C. albicans* gets changed due to presence of *Lactobacillus*. Expression of genes responsible for adherence and yeast to hyphal formation is reduced. Presence of *Lactobacillus* can alter the host immune response in case of *Candida* colonization to attract granulocytes and promote the immune defense. The picture was adapted from Bradford and Ravel [22].

Commensal bacteria generate metabolic byproducts to support their persistence in the host and confer a survival advantage over invading pathogens [32]. *Lactobacillus* spp. produce lactic acid, acetic acid, H_2_O_2_, biosurfactants and other compounds (see **Figure 1**).

### Lactic Acid

Lactobacilli produce different short chain aliphatic organic acids, like lactic acid or acetic acid. The content of acetic acid in the vaginal environment is low, ranging from 1-4 mM [101] as it is mainly produced under aerobic conditions and the vaginal environment is anaerobic or microaerobic. Indeed, acetate concentration may rise during BV [102]. Lactic acid, on the other hand, is produced through anaerobic respiration and is thought to decrease the pH in the vaginal tract [12, 103]. Domination of the vaginal microflora by *Lactobacillus* spp. is accompanied by a low pH (pH 3.5 - 4.5) [104]. Lactic acid concentration in the vaginal milieu is around 110 mM [104]. Lactic acid in combination with low pH was shown to inhibit *C. albicans* [105]. No inhibition of *C. albicans* [14, 17, 106] or *C. glabrata* [14, 17, 107] was observed at a lactic acid concentration reached with the supernatant of a cultured *L. rhamnosus* strain. The capability and rate of production of lactic acid is *Lactobacillus* strain specific. It was proposed that only elevated levels of lactic acid efficiently inhibit fungal growth [13, 107]. In support of this, supernatants of *L. rhamnosus*, *L. casei* and *L. acidophilus* exhibit an antifungal effect against *Candida* spp. only if harvested after prolonged incubation (24 h or 48 h) in which lactic acid could accumulated in the medium [13]. In addition, the reported lactic acid concentration of the vaginal tract was found to be too low to prevent growth of all relevant *Candida* species (our unpublished observation). However, in the local vaginal micro milieu or biofilm, in which higher concentrations of lactic acid could be reached, lactic acid could be a relevant antifungal agent. Also, low pH itself plays a minor role in *Candida* infections as the vaginal pH does not change during VVC in contrast to bacterial dysbiosis [5, 108]. Most studies also show that *Lactobacillus* remains the dominant bacterial species during VVC [108, 109]. The composition of the *Lactobacillus* strains differs during *Candida* infections [108, 110], which could lead to a decrease of lactic acid concentration and possibly other metabolites which in turn may allow *Candida* growth in the vaginal tract.

Stress response to weak organic acids like lactic acid differs between *Candida* species. A *C. albicans* transcriptome analysis of a set of different weak organic acids like lactic, acetic, propionic, and butyric acid led to a discovery of a complex core transcriptional response to all tested organic acids. In general, ribosomal RNA and RNA synthesis was reduced, indicating that *C. albicans* enters a starvation-like metabolic state after prolonged exposure to organic acids with reduced transcription, translation and growth. Furthermore, intracellular iron was decreased upon weak organic acid exposure [111]. Generally, exposure to weak acids leads to dramatic changes of gene expression. The involved pathways differ between species. For example, CaMig1, a transcription factor associated with glucose repression, was discovered as a central regulator of weak organic acid like lactic, acetic, propionic, and butyric acid resistance in *C. albicans,* however, it is only active in the presence of glucose [112]. In fact, glucose is limited in the vaginal tract [113], making this glucose-dependent response unlikely to contribute to lactic acid resistance in the vaginal environment. Mnl1, the *C. albicans* homologue of the yeast Com2, was found to be required for acetic acid response [114]. In *S. cerevisiae*, the transcription factors Msn2, Msn4 and War1 signal weak acid stress (e.g. sorbic acid) [115, 116]. Activation of War1 leads to expression of an ABC (ATP binding cassette) transporter gene *PDR12*, which is required for weak acid ion efflux [115] and also induced during lactic acid stress in *S. cerevisiae* [117]. Interestingly, in *C. glabrata* the high osmolarity glycerol (HOG) pathway instead of the homologs CgMsn2 and CgMsn4 is activated by sorbic acid [116]. The *C. glabrata* HOG pathway is signaling general weak acid response and osmotic and oxidative stress [116]. Deletion of *HOG1* in *C. glabrata* led to a susceptibility against lactic acid at physiological conditions. Therefore, *HOG1* response is needed for resistance to lactic acid stress [118].

Lactic acid itself may indirectly support antifungal therapy. Lactic acid and acetic acid at physiological concentrations increase efficacy of different azoles against *C. albicans*. Higher concentrations also improved efficacy of azoles against *C. glabrata* [14]. Undissociated organic acids like for example lactic acid or acetic acid lead to perturbation of plasma membrane structures in yeast cells, which may increase uptake of azoles into the yeast cell [119]. The overall concentration of organic acids like acetic acid or lactic acid may be too low to have a fungistatic effect on their own. However, facilitating azole efficacy by lactic acid could improve traditional treatment of *Candida* infections.

Anyhow, outside of the host environment, lactic acid could be less important as antifungal agent. In a co-culture system with *C. albicans, L. paracasei [120]* and *L. rhamnosus*, *L. casei* and *L. acidophilus* [13] do not acidify the environment significantly. This could indicate that these isolates do not produce sufficient amount of lactic acid. However, in contrast to these studies we find consistently strong acidification in co-cultures of *C. albicans*, *C. glabrata* with *L. fermentum*, *L. rhamnosus* or *L. gasseri in vitro* (our unpublished observations). These differences could arise from the media used for co-culture. Whereas both studies used brain heart infusion (BHI) broth for their assays [13, 120], we used MRS, which favors *Lactobacillus* growth and is slightly acidic. Of note, BHI medium is buffered to a neutral pH. *C. albicans* can utilize lactate as carbon source and can even form biofilms [121]. The *Lactobacillus* spp. generated lactate is used up by *C. albicans* as carbon source at neutral pH, explaining why in these studies *Lactobacillus* did not acidify the neutral BHI medium. This notion is also supported by a study from Willems *et al.* [122] in which a *Streptococcus mutans – C. albicans* biofilm had a higher lactic acid production, accompanied with a higher pH compared to a sole bacterial biofilm, hinting that *C. albicans* uses the lactate and thus, prevents acidification of its environment.

Taken together, lactic acid is most probably not the sole antifungal agent produced by *Lactobacillus* spp. Neutralized supernatants of *L. pentosus* [123, 124], *L. rhamnosus GR-1 and L. reuteri RC-14* [11] were able to inhibit growth of *C. albicans* [123, 124] and *C. glabrata* [11]. In support of this, inhibition of *Candida* spp. by *L. crispatus*, *L. gasseri* and *L. vaginalis* is not correlated to lactic acid production [125].

### Hydrogen Peroxide

Production of H_2_O_2_ is an important feature of *Lactobacillus* spp. to defend against bacterial infections [109, 126]. This is probably not strictly the case for *Candida* infections. The non-H_2_O_2_ producer *L. rhamnosus* GR-1 and the H_2_O_2_ producer *L. reuteri* RC-14 both inhibited growth of *C. albicans* [105]. Similar results were obtained with *C. glabrata* [11]. Most isolates of *C. glabrata* also have high tolerance to reactive oxygen species (ROS) such as H_2_O_2_ [127]. Several facts suggest that H_2_O_2_ only plays a minor role in *Candida* defense of *Lactobacillus* species in the microaerobic vaginal environment. *Lactobacillus* spp. produce H_2_O_2_ predominantly under aerobic conditions, but the conditions in the vagina are hypoxic [27]. The physiological concentration reached in *Lactobacillus* cultures (< 100 μM) does not harm lactobacilli, BV - associated bacteria and *Candida* spp. [128]. High concentrations of H_2_O_2_ (10 mM) which could harm *Candida* spp. were shown to be harmful to vaginal *Lactobacillus* species [128].

### Other antifungal factors

Other suggested antifungal factors produced by bacteria are small molecules like bacteriocins and biosurfactants [129]. Bacteriocins are proteinaceous, bacterial substances, which are able to inhibit growth of same or closely related species. Bacteriocin-like substances are very similar to bacteriocins, but often inhibit a broader range of species like gram-positive, gram-negative bacteria or fungi [130]. Pentocin TV35b is a bacteriocin-like peptide produced by *L. pentosus* which was found to have a fungistatic effect on *C. albicans* [18]. It remains the only reported bacteriocin-like peptide till today.

Adhesion to the mucosa is generally seen as the first step of infection [131, 132]. *Lactobacillus* spp. produce biosurfactants reducing adherence of competing organisms to the epithelial cell wall [133]. Some biosurfactants are active against *C. albicans* [15, 98]. For example, biosurfactants produced by *L. jensenii* and *L. gasseri* are able to reduce biofilms on polystyrene plates of *C. albicans*, *C. tropicalis* and *C. krusei* by 25%-35 [15]. CV8LAC, a biosurfactant produced by *L. brevis* is able to decrease *C. albicans* adhesion and biofilm formation to precoated medical-grade silicone [134]. The use of this biosurfactants could be developed further as a potential new coating material for medical devices to minimize *Candida* infections [134]. In general, supernatants of different *Lactobacillus* species were found to reduce adhesion of *C. albicans* to HeLa cells *(L. crispatus*, *L. gasseri*) [125], to plastic surface (*L. paracasei*) [135], as well as to TR146 cells (*L. rhamnosus*) [136]. The question remains open, if these supernatants contain unrecognized biosurfactants or if other metabolites are able to reduce adhesion.

*C. albicans* adheres to vaginal epithelial cells and initiates morphological changes of the cells leading to induction of cellular endocytosis. Treating these infected cells with *L. crispatus* lead to a decrease in adhesion, hyphal formation and proliferation of *Candida* [137]. *C. albicans* adhesion to Vk2/E6E7 cells was reduced by their preincubation with extracellular polysaccharide (EPS) produced by *L. crispatus* L1 [16]. This reduction was similar to the cell-dependent reduction of adhesion by a preincubated *L. crispatus* L1 [16]. However, co-cultivation of EPS and *C. albicans* on Vk2/E6E7 cells did not reduce adhesion of *C. albicans*, whereas co-culture of *C. albicans* and *L. crispatus* lead to a reduction of adhesion of *C. albicans* [16]. EPS could therefore be a putative new coating agent. The cell-dependent reduction of adhesion is probably due to co-aggregation of *Lactobacillus* and *Candida* species. Co-aggregation is a characteristic of early biofilm formation as it involves adhesion-receptor interactions between the microbial cell surfaces. Therefore, competition for binding sites could be partly influencing proper adhesion of *Candida* to mucosal surfaces [12]. With regard to studies with NAC species, *L. reuteri* [12], *L. pentosus* [123] *L. rhamnosus* GR-1 and *L. reuteri* RC-14 [11] were shown to possess the ability to co-aggregate with various *Candida* species besides *C. albicans*, for example *C. glabrata [11, 12]*, *C. krusei* [12] or *C. tropicalis* [123]. Interestingly, good initial adhesion of *L. gasseri, L. crispatus* or *L. vaginalis* isolates was not consistent with good inhibition of adherence of *C. albicans* [125]. This suggests that minimizing the adhesion of *C. albicans* is not solely due to saturation of adhesion sites, but rather through either changes in the epithelial cell surface or due to influencing the adhesion ability of the pathogen itself [125].

*Lactobacillus* spp. influence *C. albicans* morphology. Hyphae formation was impaired in co-culture with *L. paracasei* [120, 135]. Interaction of *Lactobacillus* and *C. albicans* alters the gene expression pattern towards the yeast form. *C. albicans* yeast form generally shows reduced adhesion and biofilm formation [54]. Interaction between *Lactobacillus* spp. and *Candida* leads to expression changes of genes associated with biofilm formation, yeast to hyphal transition and adhesion. For example, *ALS3*, *EFG1* or *HWP1*, were suppressed in *C. albicans* cells treated with *L. paracasei* supernatant [135, 138]. Efg1 is a regulator for several genes responsible for yeast-hyphae transition such as *ALS3*, *HWP1* or SAP (secreted aspartate proteases) genes [139, 140]. Furthermore, interaction with *L. paracasei* induced expression of *YWP1*, a gene associated with the yeast form [135]. Furthermore, *PHR1,* a pH responsive gene coding for a glucan remodeling enzyme supporting hyphal growth in *Candida,* was downregulated in *C. albicans* co-cultured with L. *reuteri* RC-14 and *L. rhamnosus* GR-1 [105, 141]. This suggests that *Lactobacillus* spp. influences *C. albicans* to stay in its less invasive form, which could help preventing overgrowth of the fungus. In addition, the interaction directly leads to downregulation of several *C. albicans* genes related to adhesion, invasion and counteraction of host defenses [138]. In *C. glabrata* altered gene expression of genes related to adhesion in the presence of *L. rhamnosus* and *L. reuteri* supernatants was also observed. Downregulation of the adhesion gene *YAK1* was accompanied by reduced levels of the Yak1-dependent adhesin *EPA6*, which is involved in adhesion and biofilm development [107, 142]. This indicates that presence of *Lactobacillus* spp. decreases adhesion and maybe virulence of *C. glabrata*.

RNA-Seq of *C. albicans* gene expression in response to a TR146 cell monolayer, which was preincubated with *L. rhamnosus GG* revealed upregulation of genes involved in fatty acid catabolism, glyoxylate cycle and gluconeogenesis and downregulation of glycolysis and ergosterol biosynthesis genes [136]. Another study investigating *L*. *reuteri* RC-14 and *L. rhamnosus* GR-1 co-culture found an upregulation of glycolysis and a reduced expression of genes relating to gluconeogenesis [105]. Thus, strain-specific effects are obviously an important factor to keep in mind while exploring *Lactobacillus* spp. as potent novel probiotics. Furthermore, one study was conducted on cell layer surface, while the other one on an abiotic surface [105, 136]. This could be an indicator for the importance of proper *in vitro* systems, mimicking the actual environment as close as possible.

### Three-way interactions including the host

The ability to recognize and sense a pathogen is crucial for the immune system to initiate an immune response. *Candida* spp. have a cell wall, consisting of carbohydrate polymers such as mannans, β-glucans and chitin merged with a protein matrix [143]. This cell wall components can be detected by Toll-like receptor family (TLR), C-type lectin receptor (CLRs) family like Dectin-1, Dectin-2 or Mincle, Galectin-3 and scavenger receptors [143] which start the immune response. For example, *C. albicans* mannans gets recognized by TLR4, whereas β-glucans are sensed by Dectin-1 [143]. CLRs are the responsible receptor family for the immune reaction against disseminated Candidiasis [144]. CBL-B, an E3-ubiquitin ligase, controls availability of Dectin-1 and -2 receptors in phagocytes [144]. Interestingly, i*n vivo* testing in mice showed that inhibition of CBL-B conveys a protective effect against *C. albicans* systemic infections [144]. Single nucleotide polymorphism (SNP) in different TLRs also can lead to increased susceptibility against *C. albicans* and Candidiasis, for example SNP in TLR1 is associated with an impaired cytokine release during *C. albicans* infection [143].

Activation of the host innate immune response by *C. albicans* leads to the production of various cytokines and chemokines by epithelial cells [145]. An effective Th1 response is crucial for defense against *C. albicans* infections [20, 145, 146]. Development of antifungal Th1 response is initiated by TLRs [143]. Th2-type response, on the other hand, is considered as nonprotective against the fungi [147, 148]. Th1 response comprises production of INF-γ, TNF β IL-6 and IL-2, which are protective against most fungal infections [149]. Humoral immunity is mediated by Th2 response, which produce IL-4, IL-5 and IL-13 [150]. Upregulation of Th2 response is associated with reduced IFN- γ production and therefore, correlating with higher disease severity and poor prognosis [149]. Th2 induction was found to be dependent on Type 1 interferon (INF-1) cytokines [151]. INF-1 are associated with mediation of lethal effects during disseminated *Candida* infections [152], which could be another explanation why Th2 response correlates with poor disease progression. Additionally, there are Th17 cells which, among others, produce IL-17. Th17 cytokines are thought to exhibit a protective role against mucosal and disseminated fungal infections [149]. Sole *C. albicans* interaction leads to a decrease of IL-2 and IL-4 production in epithelial cells. In addition, IL-17 response gets impaired, which suggests a diminished inflammatory immune response [137]. Furthermore, *C. albicans* hyphae bind to TLR2 and induce the production of IL-10, an anti-inflammatory cytokine, which leads to fewer regulatory T cells [153] and therefore a decreased host immune response. In mice, IL-10 expression is associated with higher susceptibility to candidiasis [154, 155]. IL-8 and IL-1α secretion gets significantly increased upon *C. albicans* treatment, indicating that they play an important role in the natural host's defense against the yeast infection [19].

Treatment of epithelial cells with only *Lactobacillus* spp. also alters the immune response. For example, *L. crispatus* increased IL-2 and decreased IL-8 response of vaginal epithelial cells [137]. Another study found that stimulation of epithelial cells with *L. rhamnosus* GG or *L. reuteri* RC*-*14 without prior interaction with *Candida* leads to an upregulation of IL-8 release [19, 136]. Treated cells showed neither visible damage [136] nor induction of apoptosis [156]. IL-8 acts as chemoattractant for polymorphonuclear leukocytes (PMNs) and other granulocytes. PMNs are associated with defense mechanisms against *Candida* infections [157]. Additionally, they induce a Th1 response [20]. Therefore, induction of IL-8 by *Lactobacillus spp.* could work as a protective mechanism for the host by attracting PMNs to quicken the immune response in case of an infection.

Simultaneous treatment of epithelial cells with *C. albicans* and *Lactobacillus* supernatants showed increased IL-8 (*L. reuteri* RC-14) and IFNγ-induced protein 10 (IP-10) (*L. rhamnosus* GR-1) release and reduced the inflammatory response of the host [19]. However, other studies showed that *L. plantarum 59* and *L. fermentum* interaction downregulates IL-8 response in *C. albicans* infection on HeLa cells [158]. Quantity of antifungal agents produced by *Lactobacillus* spp. like lactic acid could in turn lead to a variance in cytokine response [19]. Treatment with *L. crispatus* was able to mitigate *C. albicans*-induced reduction in IL-17 expression of vaginal epithelial cells [137]. This would indicate that *L. crispatus* prevents *C. albicans* from downregulating an IL-17 dependent immune response. However, the role of Th17 response in VVC is not fully clear yet [159, 160]. Cells preincubated with *L. rhamnosus* have decreased release of lactate dehydrogenase (LDH) during infections [136]. LDH is a soluble enzyme, found in almost every living human cell and is responsible for lactic acid fermentation. In case the cell membrane is damaged, it is released into their surrounding extracellular space and serves as a cell death marker [161]. Decreased release of LDH suggests that cells, treated with *Lactobacillus* spp., are protected from *C. albicans* induced cell damage [136]. Another way how *Lactobacillus* spp. could mediate tolerance to *C. albicans* on the mucosa is by producing tryptophan catabolites via Indolamin-2,3-Dioxygenase *IDO1* [162]. These act on regulatory T-cells which results in raising local expression of IL-22 and thus, could provide immunoprotection to VVC. *IDO1* and IL-22 deficiency in animals is linked to increased susceptibility in VVC [163].

The host immune response in *C. glabrata* infection is generally lower than with *C. albicans* [164]. It was shown that only granulocyte macrophage colony-stimulating factor (GM-CSF) is induced by *C. glabrata.* GM-CSF is a potent activator of macrophages and initiates recruitment of macrophages [164]. Since *C. glabrata* has the ability to survive and replicate in macrophages, it could be that the fungus attracts macrophages on purpose [164]. There are no studies addressing the immune response towards *C. glabrata* or other NAC species in presence of *Lactobacillus* spp.

Interestingly, treatment of vaginal cells with lactic acid (33 mM) decreases the production of IL-6 and IL-8 and significantly increases production of the anti-inflammatory cytokine IL-1 receptor antagonist (IL-1RA) which reduces the inflammatory activity of IL-1α and IL-1β [165]. This suggests that lactic acid alone is sufficient to decrease production of pro-inflammatory molecules. However, it is currently unclear if this repression can be obtained when treating the cells with *Candida* and lactic acid simultaneously.

## CONCLUSION

*Lactobacillus* species are promising candidates to improve treatment of vulvovaginal *Candida* infections. Results often vary between *Lactobacillus* and *Candida* strains, making it difficult to pinpoint specific pathways and mechanisms. The probiotic effect seen *in vitro* of *Lactobacillus* strains is probably only partly due to the accumulation of lactic acid. The lactic acid content in the vaginal tract is too low to have an effect on *Candida* spp. and one has to assume that higher local concentrations of lactic acid are possibly preventing overgrowth of *Candida* spp. in close proximity to *Lactobacillus*. In addition, organic acids produced by *Lactobacillus* spp. do positively influence efficacy of antifungal agents by increasing the permeability of fungal plasma membrane structure, which facilitates azole uptake. Still, lactic acid, low pH and, to a minor extent, other secreted metabolites are environmental signals sensed by *C. albicans* and are leading to changes of gene expression and transition to hyphal growth. Another putative mechanism for the probiotic effect might be competition for available niches and reduced adhesion. All in all, the antifungal effect **(Figure 1)** of *Lactobacillus* comprises different aspects and has a species and strain dependent components. So far, most studies concentrated on *C. albicans*. However, NAC species have usually different resistance profiles and may also require different treatments. Moreover, studies addressing these fungi are still scarce. Further investigations are definitely needed to expand our knowledge on NAC*-Lactobacillus* interactions. All in all, carefully selected *Lactobacillus* species active against specific *Candida* species could lead to improved treatment options.

## Abbreviations:

BV: – bacterial vaginosis,
CLR: – C-type lectin receptor,
EPS: – extracellular polysaccharide,
GM-CSF: – granulocyte macrophage colony-stimulating factor
IFN: – interferon,
LDH: – lactate dehyrogenase,
NAC: – non-albicans Candida,
PMN: – polymorphonuclear leukocyte,
SNP: – single nucleotide polymorphism,
TLR: – Toll-like receptor,
VVC: – vulvovaginal candidiasis.
